# Identification of primary sclerosing cholangitis: ICD-10 code validation and comparison with a large language model approach

**DOI:** 10.21203/rs.3.rs-9152968/v1

**Published:** 2026-04-01

**Authors:** Melinda Wang, Mai Dao, Molly Delk, Cynthia Fenton, Michelle Y. Li, Jessica B. Rubin, Jin Ge, Jennifer C. Lai, Michael Li

**Affiliations:** University of California-San Francisco; University of California-San Francisco; Tulane University; University of California-San Francisco; University of California-San Francisco; University of California-San Francisco; University of California-San Francisco; University of California-San Francisco; University of California-San Francisco

**Keywords:** primary sclerosing cholangitis, algorithm, ICD, database, artificial intelligence

## Abstract

**Background:**

Retrospective studies investigating primary sclerosing cholangitis (PSC) have been limited by the absence of a PSC-specific diagnostic code. In 2018, a new PSC-specific ICD-10 code was introduced.

**Aims:**

We aimed to validate the new ICD-10 code and compare it to other methods of identifying patients with PSC.

**Methods:**

All gastroenterology/hepatology and primary clinic notes and discharge summaries were extracted from UCSF Epic Clarity database and potential PSC patients were identified using natural language processing (NLP). PSC diagnosis was determined by physician adjudication through chart review. LASSO regression was used to develop and internally validate a PSC prediction model. Separately, we tested large language model’s (LLM) ability to distinguish PSC from non-PSC patients.

**Results:**

Among 867 patients identified using NLP, 226 (26%) patients were adjudicated to have a true PSC diagnosis. The LASSO model selected ICD-10 code, alkaline phosphatase > 120 IU/L, ursodiol use, inflammatory bowel disease, and history of cholangitis. ICD-10 code alone had a c-statistic of 0.87, sensitivity 87.6%, and PPV 68.8%. The LASSO model had a c-statistic of 0.92, sensitivity 87.4%, and PPV 70.7%. LLM had a c-statistic 0.77, sensitivity 91.7%, and PPV 51.0%.

**Conclusions:**

The PSC-specific ICD-10 code had excellent discriminatory capacity for identifying patients with PSC. While an optimized PSC prediction algorithm had slightly improved test characteristics, ICD-10 code alone was sufficient in identifying patients with PSC, supporting the use of the ICD-10 code in future database studies of PSC. In contrast, LLM had inferior discrimination compared to either ICD-10 code or the prediction model.

## Introduction

Primary sclerosing cholangitis (PSC) is a rare, progressive cholestatic liver disease that causes inflammation and fibrosis of the biliary tree, often leading to cirrhosis and need for liver transplantation.^1–8^ Patients with PSC can develop life-threatening complications such as bacterial cholangitis and malignancies, including cholangiocarcinoma and other gastrointestinal-related cancers.^1–3^ PSC is associated with high healthcare utilization and often poor health-related quality of life.^4–8^ Unfortunately, there are currently no effective therapies that alter the natural history of PSC.^1–3^ Large databases can be helpful to study rare diseases such as PSC (which has a prevalence of 0–31.7 per 100,000 persons)^9^, however, prior diagnostic codes for PSC have not been specific and encompassed other biliary diseases like acute cholangitis.^10^ Thus, prior PSC-related studies have depended on single-center experiences with cases often adjudicated by manual chart review.^10–13^

Recognizing a need for a PSC-specific code, an ICD-10 code for PSC was introduced in October 2018 (ICD-10 K83.01). Newer administrative database studies using the new PSC-specific ICD-10 code demonstrate similar demographic characteristics to prior epidemiological studies.^14, 15^ The new PSC-specific ICD-10 code, however, has not yet been validated.^8^ With recent technological advancements, artificial intelligence systems such as large language models (LLMs) are also reshaping the landscape of clinical research. By leveraging deep learning techniques, LLMs can extract and synthesize disease-related information from unstructured electronic health record text, substantially enhancing the analytical power and efficacy of clinical research. Recent studies have demonstrated that LLMs can outperform ICD-10 codes in identifying disease from unstructured text.^16, 17^ Beyond simple disease labeling, LLMs offer the potential to capture nuanced clinical features and generate probabilistic differential diagnoses directly from narrative notes. This capacity positions LLMs as a powerful complementary approach to structured coding systems in disease identification and prediction. Given these rapid advances, we sought to validate the new PSC-specific ICD-10 code, to develop a prediction model for PSC including the ICD-10 code, and to compare the discrimination of the ICD-10 code alone to the prediction model using clinical data, and to processing by a large language model (LLM).

## Methods

Gastroenterology, hepatology and primary care clinic notes and discharge summaries between 10/1/2018 (when the new PSC ICD-10 code was introduced) and 6/1/2023 for patients ≥ 18 years were extracted as unstructured data from the UCSF Clarity Clinical Data Warehouse database. All patients with the terms “PSC” or “primary sclerosing cholangitis” within the text were identified using natural language processing (NLP) with regularized expressions. Notes with “PSC” embedded within text, preceded by “does not have,” “without,” “no,” “not,” “denies,” or “rule out,” and followed by “also increases risk” were excluded. All patients with the new PSC ICD-10 code (K83.01) were also identified and included in the data extraction. Clinical data including maximum laboratory values (alkaline phosphatase, IgG4, AMA, ANA, IgG, SMA, SLA), magnetic resonance cholangiopancreatography (MRCP) imaging reports, endoscopic retrograde cholangiopancreatography (ERCP) procedure reports, medications (e.g., ursodiol), additional diagnoses by ICD-10 code (inflammatory bowel disease (IBD), autoimmune hepatitis, primary biliary cholangitis, acute cholangitis, cirrhosis, colorectal cancer, cholangiocarcinoma) and pathology reports from liver biopsies were extracted for all patients from the Clarity database (Supplemental Table 1). Three physicians (MW, MD, MD), who were blinded to the presence/absence of the PSC ICD-10 code, reviewed the extracted data to adjudicate the presence or absence of PSC for each patient. A true PSC diagnosis was defined as an elevated alkaline phosphatase (> 120 IU/mL) and either radiographic (MRCP), endoscopic (ERCP), and/or biopsy evidence of PSC. For patients who did not meet criteria for PSC as defined above but had documentation from a gastroenterologist or hepatologist confirming a PSC diagnosis (e.g., MRCP or liver biopsy performed at an outside institution and therefore not able to be extracted automatically from the Clarity database) (n = 36), an attending hepatologist (ML) provided final adjudication of presence/absence of PSC diagnosis.

For the prediction model, the dataset was split randomly into a training set (70%) and a test set (30%). We constructed a LASSO regression model using the following covariates: ICD-10 code, elevated alkaline phosphatase, ursodiol treatment, and the presence of inflammatory bowel disease, primary biliary cholangitis, autoimmune hepatitis, and/or bacterial cholangitis based on compatible ICD-10 codes. We utilized 10-fold cross-validation to determine the optimal lambda regularization parameter (using the 1-standard error rule).^18^ The resulting model was then internally validated in the independent test set. To enable calculation of sensitivity and positive predictive value (PPV), the optimal cutoff for the LASSO regression model was calculated by maximizing Youden’s index (the j-statistic).

We also tested the ability of a large language model (LLM) to detect and confirm the presence of PSC in clinical notes. For this exercise, we used protected health information (PHI)-compliant versions of the Microsoft Azure OpenAI Generative Pre-Trained Transformer-4 Omni (GPT-4o) available at our institution. These models are deployed within UCSF’s secure “Versa” platform, which has been previously described and used to analyze clinical documentation in hepatology.^16^ GPT-4o was prompted in a zero-shot fashion (e.g., no example instructions or answers were given to the LLM). We iteratively refined the prompt until the LLM’s accuracy was greater than 90% in a random 5% sample of the total number of notes identified by NLP. The final prompt used is provided in the supplemental materials (Supplementary Text 1). Finally, the c-statistic, sensitivity, and PPV were calculated for the ICD code alone, the LASSO regression model, and the LLM.

## Results

In total, 867 patients were identified through NLP to have (1) an inpatient or outpatient note with the term “PSC” or “primary sclerosing cholangitis”, and/or (2) the PSC ICD-10 code within the study period. Of these patients, 288 (33%) had the PSC ICD-10 code. After physician review of laboratory, radiographic, and biopsy data, 226 (26%) patients were adjudicated to have true PSC. Table 1 displays clinical characteristics comparing the PSC (n = 226) and non-PSC (n = 579) groups in our study. Compared to non-PSC patients, PSC patients were younger (44 vs 52 years, p < 0.01) and had greater prevalence of PSC-related conditions including cirrhosis (61% vs 44%, p < 0.01), inflammatory bowel disease (76% vs 44%, p < 0.01), acute cholangitis (97% vs 42%, p < 0.01), and cholangiocarcinoma (11% vs 4%, p < 0.01). PSC patients also had significantly higher alkaline phosphatase levels (550 vs 273, p < 0.01) and were significantly more likely to have a history of ursodiol treatment (75% vs 41%, p < 0.01).

The ICD-10 code alone had a sensitivity of 87.6% (198/226; 95%CI 82.6–91.6) and a positive predictive value of 68.8% (198/288; 95%CI 63.1–74.1). The c-statistic for the ICD-10 code as a solitary predictor of PSC was 0.87 (95%CI 0.84–0.89).

LASSO regression eliminated PBC and AIH from the final model (i.e., the coefficients for PBC diagnosis and AIH diagnosis were reduced to zero). Based on the LASSO regression coefficients, the probability of having a true PSC diagnosis was defined as follows:

$$\text{Probability}\;\text{of}\;\text{having}\;\text{true}\;\text{PSC}=1/\left[1+e-\left(-3.54+\left(2.76*\text{ICD}\;\text{code}\right)+\left(0.41*\text{alkaline}\;\text{phosphatase}\right)+\left(0.27*\text{ursodiol}\right)+\left(0.31*\text{ibd}\right)+\left(0.53*\text{cholangitis}\right)\right)\right]$$

The c-statistic in the test set was 0.92 (95%CI 0.90–0.95). On internal validation, there was minimal model optimism (c-statistic in the test set 0.91 (95%CI 0.87–0.95)). The optimal cutpoint identified by maximizing Youden’s index was 0.50 (i.e., the model estimating a 50% probability of a true PSC diagnosis). Using this dichotomized cutoff, the LASSO regression model had a sensitivity of 87.4% (195/223; 95%CI 82.4–91.5%) and positive predictive value of 70.7% (195/276; 95%CI 64.9–76.0). Figure 1 shows ROC curves for ICD-10 only and PSC prediction model.

LLM alone had a sensitivity of 91.7% (187/204; 95%CI 87.0–95.1) and a positive predictive value of 51.0% (187/367; 95%CI 45.7%−56.2). C-statistic for LLM alone was 0.77 (95%CI 0.75–0.81). Table 2 shows test characteristics for the ICD-10 code alone, the LASSO regression model, and the LLM.

## Discussion

Prior identification of patients with PSC in administrative datasets required the use of multiple ICD-9 codes, including a cholangitis-specific ICD code and an IBD-related ICD code.^10, 19^ Despite the use of multiple diagnostic codes to create an optimal algorithm, this method only had a sensitivity of 56% and positive predictive value of 59%.^10^ The ICD-9 code for cholangitis alone had a sensitivity of 83.7% and PPV of 7.2%.^10^ These prior data suggested that with the ICD codes available, it was not possible to accurately identify patients with PSC.

With the development of a new ICD-10 code specific to PSC, we observed markedly improved sensitivity and positive predictive value using the ICD-10 code alone as a predictor of PSC. We investigated whether we could optimize an algorithm for predicting PSC by including additional variables that we considered *a priori* to be associated with a true PSC diagnosis (e.g., elevated alkaline phosphatase, ursodiol treatment, concurrent inflammatory bowel disease, etc.). Though we developed and internally validated a prediction model with excellent discrimination, the model’s performance was not meaningfully improved over the ICD-code alone (c-statistics of 0.91 vs 0.87, sensitivities of 87.9% vs 87.6%, and PPVs of 70.3% vs 68.8%, respectively. Based on our data, it seems clear that the ICD code alone is sufficient for use in database-related research.

Notably, the LLM yielded substantially lower sensitivity and discrimination. Compared to ICD-10 codes that are more inclusive for billing purposes, our prompting of the LLM required explicit documentation of PSC to generate a positive prediction. This likely explains GPT-4o’s conservative classification compared to ICD-10 code alone. In addition, in our implementation, the LLM was limited to evaluating unstructured text in clinic notes but did not have access to other structured (i.e. laboratory tests, diagnostic codes) and unstructured (i.e. MRCP reports, ERCP reports, pathology reports) that adjudicators in this study used to determine the presence/absence of PSC. Providing these data to LLM may improve LLM performance but given that ICD-10 code alone demonstrated strong discrimination and is easier to extract from the electronic health record, the added complexity of feeding multiple structured and unstructured data elements did not appear justified.

The PSC prediction model included additional variables including alkaline phosphatase, ursodiol use, and associated diagnoses including IBD and acute cholangitis. The prevalence of IBD in the PSC patients included in this study was 76%, in line with the reported prevalence of 70–80% in the general PSC population.^20^ In addition, acute bacterial cholangitis is a classic complication among patients with PSC.^21^ Ursodiol was also incorporated in the optimal PSC prediction model – this is because while there are no current therapies to treat PSC, many patients with PSC are prescribed ursodiol.^3^ Studies demonstrate that while ursodiol may improve liver tests, ursodiol does not improve survival and may lead to increased rates of adverse events.^22^ With new potential therapies for PSC on the horizon,^23^ rates of ursodiol use among patients with PSC may decrease, making this variable less effective in a PSC prediction model. Finally, autoimmune hepatitis and PBC were eliminated by LASSO regression. The prevalence of autoimmune hepatitis overlap in our PSC cohort (15%) is similar to what has been reported in the general PSC population (7–14%) though notably the “prevalence” of PBC overlap (58%) far exceeds case reports in the literature.^24^ As these diagnoses were made based on ICD-10 codes, we strongly suspect that most PSC patients in our cohort experienced miscoding for one or more billable events (e.g., the PBC diagnosis code was inputted instead of the PSC code) leading to the very high proportion of PSC patients also carrying a PBC diagnosis code. Furthermore, our data showing expected prevalence of IBD and autoimmune hepatitis in our PSC cohort supports the validity of our approach to validate true PSC diagnoses.

PSC has been recognized as a spectrum of disease ranging from small duct PSC to classic, large duct PSC. While patients with large duct PSC have classic diagnostic features to assist with diagnosis, small duct PSC, a rarer form of PSC with better prognosis, can often be challenging to diagnosis.^25^ The definition of PSC in this population included classic diagnostic features of large duct PSC and also hepatologist/gastroenterologist statement of PSC even without known diagnostic features. This definition attempts to capture patients with PSC across the spectrum of disease. However, given diversity of PSC presentations, future studies may benefit from evaluating the validity of the ICD-10 code within a larger and more diverse administrative dataset.

This study has several limitations. First, data were limited to a single center experience which does not capture different coding practices across systems. Second, variables used within the PSC optimization model were limited by variables available in the electronic health record. Finally, the study cohort included patients with positive ICD-10 code for PSC and patients identified in gastroenterology/hepatology and primary care notes or discharge summaries to have “PSC” or similar iterations in the notes by national language processing. Thus, negative predictive value could not be calculated within this study population and positive predictive value must be interpreted with caution as the population is enriched with patients likely to have PSC which would artificially inflate the positive predictive value.

In conclusion, we validated the new PSC-specific ICD-10 code and demonstrated use of either the ICD-10 code alone or an optimized PSC prediction model including additional clinical variables are effective in identifying patients with PSC in administrative databases. Further, an LLM approach solely using unstructured data from notes was inferior. Future studies can safely utilize the ICD-10 code for investigating PSC.

## Supplementary Material

Table 1

Table 1 is available in the Supplementary Files section.

## Figures and Tables

**Table 2:** Test Performance of ICD only, LASSO regression model, and large language model for identifying PSC

C-statistic	ICD-code only	LASSO Regression Model	Large Language Model
	0.87 (0.84–0.89)	0.92 (0.90–0.95)	0.77 (0.75–0.81)
Sensitivity	87.6 (82.6–91.6)	87.4 (82.4–91.5)	91.7 (87.0–95.1)
Specificity	86.0 (83.0–88.6)	86.4 (83.4–89.1)	63.8 (59.4–68.0)
Positive Predictive Value	68.8 (63.1–74.1)	70.7 (64.9–76.0)	51.0 (45.7–56.2)

## Abbreviations

PSC: primary sclerosing cholangitis
NLP: national language processing
PPV: positive predictive value
AMA: anti-mitochondrial antibody
ANA: anti-nuclear antibody
SMA: smooth muscle antibody
SLA: soluble liver antigen antibody
MRCP: magnetic resonance cholangiopancreatography
ERCP: endoscopic retrograde cholangiopancreatography
IBD: inflammatory bowel disease
PHI: patient health information
